# Risk analysis of inter-species reassortment through a Rift Valley fever phlebovirus MP-12 vaccine strain

**DOI:** 10.1371/journal.pone.0185194

**Published:** 2017-09-19

**Authors:** Hoai J. Ly, Nandadeva Lokugamage, Shoko Nishiyama, Tetsuro Ikegami

**Affiliations:** 1 Department of Pathology, The University of Texas Medical Branch, Galveston, Texas, United States of America; 2 Sealy Center for Vaccine Development, The University of Texas Medical Branch, Galveston, Texas, United States of America; 3 Center for Biodefense and Emerging Infectious Diseases, The University of Texas Medical Branch, Galveston, Texas, United States of America; George Mason University, UNITED STATES

## Abstract

Rift Valley fever (RVF) is a mosquito-borne zoonotic disease endemic to Africa and the Arabian Peninsula. The causative agent, Rift Valley fever phlebovirus (RVFV), belongs to the genus *Phlebovirus* in the family *Phenuiviridae* and causes high rates of abortions in ruminants, and hemorrhagic fever, encephalitis, or blindness in humans. Viral maintenance by mosquito vectors has led to sporadic RVF outbreaks in ruminants and humans in endemic countries, and effective vaccination of animals and humans may minimize the impact of this disease. A live-attenuated MP-12 vaccine strain is one of the best characterized RVFV strains, and was conditionally approved as a veterinary vaccine in the U.S. Live-attenuated RVF vaccines including MP-12 strain may form reassortant strains with other bunyavirus species. This study thus aimed to characterize the occurrence of genetic reassortment between the MP-12 strain and bunyavirus species closely related to RVFV. The Arumowot virus (AMTV) and Gouleako goukovirus (GOLV), are transmitted by mosquitoes in Africa. The results of this study showed that GOLV does not form detectable reassortant strains with the MP-12 strain in co-infected C6/36 cells. The AMTV also did not form any reassortant strains with MP-12 strain in co-infected C6/36 cells, due to the incompatibility among N, L, and Gn/Gc proteins. A lack of reassortant formation could be due to a functional incompatibility of N and L proteins derived from heterologous species, and due to a lack of packaging via heterologous Gn/Gc proteins. The MP-12 strain did, however, randomly exchange L-, M-, and S-segments with a genetic variant strain, rMP12-GM50, in culture cells. The MP-12 strain is thus unlikely to form any reassortant strains with AMTV or GOLV in nature.

## Introduction

Vaccination has led to many public health accomplishment such as the eradication of smallpox or near eradication of poliomyelitis [1]. Many viral diseases have not, however, been successfully prevented by vaccination due to a lack of either vaccines or the coverage of vaccination in susceptible populations. The *Bunyavirales* order consists of nine RNA virus families, encompassing more than 150 species and 13 genera, including highly pathogenic species, which lack effective vaccines for the prevention of outbreaks. Rift Valley fever (RVF), caused by *Rift Valley fever phlebovirus* (RVFV: genus *Phlebovirus*, family *Phenuiviridae*), is one of the most important zoonotic viral diseases in Africa and the Arabian Peninsula. RVFV causes high rates of abortions in sheep, cattle, goats, and camels, leading to devastating economic losses in the agricultural industry [2]. RVFV is also highly pathogenic to humans, as severely affected patients succumb to hemorrhagic fever, encephalitis, or retinitis [3,4]. RVFV is naturally maintained by floodwater *Aedes* mosquitoes via vertical viral transmissions [5,6], whereas horizontal transmission via other mosquito species (e.g., *Culex* spp.) likely plays an important role in viral transmission to animals or humans [6,7].

The bunyavirus genome consists of three RNA segments, the Large (L)-, Medium (M)-, and Small (S)-segments with negative or ambi-sense polarity. The co-infection of two different strains or species could lead to the generation of a reassortant strain encoding one or more RNA segment(s) derived from heterologous bunyaviruses. Between 1997 and 1998, the Ngari virus was isolated from patients with hemorrhagic fever during an outbreak of RVF in Kenya, Tanzania, and Somalia [8]. Genetic analysis of the Ngari virus revealed that the S- and L-segments were related to those of the Bunyamwera virus, and the M-segment was closely related to that of the Batai virus [9]. The nucleotide similarity between M-segments of the Batai virus and Bunyamwera virus was approximately 64%, which indicates that genetic reassortment between two bunyavirus species is a concern, as it could generate novel pathogenic bunyaviruses. Little is known about the likelihood of RVFV generating any reassortant strains with heterologous phlebovirus species. This is particularly important if a live-attenuated vaccine strain is able to alter a pathogenic phenotype via genetic reassortment. In Africa, several phleboviruses are transmitted via different vector hosts: i.e., (i) sandfly-borne phleboviruses: Sandfly fever Sicilian virus (SFSV), Sandfly fever Naples phlebovirus (SFNV), Toscana virus (TOSV), and Punique virus (PNV), (ii) mosquito-borne phleboviruses: Arumowot virus (AMTV: transmitted by *Culex antennatus*), and Odrenisrou virus (ODRV: transmitted by *Culex albiventris*), and (iii) phleboviruses isolated from wild rodents: Gabek Forest virus (GFV), Gordil virus (GORV), and Saint Floris virus (SAFV) [10–13]. *Culex antennatus* is a potent vector for both AMTV and RVFV [14], and sheep is naturally exposed to AMTV, ODRV, and RVFV in Burkina Faso [12]. Experimental infection of sheep revealed that transient fever occurs by AMTV, but not by GORV, SAFV, or GFV [15]. AMTV shares the endemic area with RVFV, and is distributed to at least Burkina Faso, Sudan, South Africa, Sierra Leone, Kenya, Niger, Morocco, and Tunisia [15,16]. Within the *Phenuiviridae* family, Gouleako goukovirus (GOLV: genus Goukovirus) is also transmitted via mosquitoes: *Anopheles*, *Culex*, and *Uranotaenia* spp. in Africa [13,16,17]. GOLV is a mosquito-restricted virus isolated in West Africa, and its replication is impaired at temperatures 31°C and above [18]. In 2013, an investigation using RT-PCR analysis suspected the presence of a GOLV-like virus in the lung tissues of pigs with severe bronchopneumonia with unknown etiology in the Republic of Korea [19]. GOLV derived from Ivory Coast did not, however, replicate in porcine kidney 15 (PK15) cells, and thus, the mechanism of GOLV-like virus infection in pigs in the Republic of Korea might be due to the unique species specificity of GOLV-like virus [20]. Characterization of genetic reassortment between RVFV and other bunyaviruses is required for the identification of the potential environmental risk upon the vaccination using live-attenuated RVF vaccines. In this study, we aimed to characterize the formation of reassortant strains between RVFV MP-12 vaccine strain and AMTV, and between MP-12 strain and GOLV. Our hypothesis is that RVFV cannot form reassortant strains with AMTV or GOLV due to a lack of compatibility among heterologous N, L, and/or Gn/Gc proteins. We utilized minigenome systems to evaluate the genetic compatibility of N, L, and Gn/Gc proteins, whereas co-infection experiments in *Aedes albopictus* C6/36 cells were performed to detect any reassortant strains.

## Materials and methods

### Media, cells, and viruses

Vero cells (ATCC CCL-81), VeroE6 cells (ATCC CRL-1586), and PK15 cells (ATCC CCL-33) were maintained at 37°C with 5% CO_2_ in Dulbecco’s modified minimum essential medium (DMEM) containing 10% fetal bovine serum (FBS), penicillin (100 U/ml), and streptomycin (100 μg/ml). BHK/T7-9 cells that stably expressed T7 RNA polymerase [21] were maintained at 37°C with 5% CO_2_ in MEM-alpha containing 10% FBS, penicillin (100 U/ml), streptomycin (100 μg/ml), and hygromycin B (600 μg/ml). C6/36 cells (ATCC CRL-1660) were maintained at 28°C without CO_2_ in Leibovitz’s L-15 medium containing 10% FBS, 10% tryptose phosphate broth (TPB), penicillin (100 U/ml), and streptomycin (100 μg/ml). Cells used in this study were verified to be mycoplasma free at the UTMB Tissue Culture Core Facility.

A recombinant MP-12 strain (rMP-12) and rMP-12 encoding an in-frame 69% deletion of the NSs gene (rMP12-ΔNSs16/198, alternative name: rMP12-C13type) have been previously described [22]. An rMP-12 variant encoding several silent mutations every 50 nucleotides within the N, NSs, M, and L open reading frames (ORFs) was recovered by reverse genetics, and was designated rMP12-GM50. In addition, reassortant rMP-12 strains encoding either the rMP12-GM50 L-segment (named RST-GM50-L), rMP12-GM50 M-segment (named RST-GM50-M), or rMP12-GM50 S-segment (named RST-GM50-S) were generated by reverse genetics for use as controls in the validation experiments for genotyping. Rescued viruses were plaque-cloned once, and then amplified twice in Vero cells after recovery from BHK/T7-9 cells. AMTV Ar 1286–64 strain (Sudan, 1963) and GOLV A5/CI/2004 strain (Ivory Coast, 2004) were kindly provided by Dr. Robert B. Tesh at the University of Texas Medical Branch at Galveston (UTMB). AMTV was plaque-cloned once, and then amplified three times in Vero cells, whereas GOLV was amplified twice in C6/36 cells at 28°C before experimental use. All stock viruses were sequenced, titrated by plaque assay, and then used for subsequent experiments.

### Plasmids

Plasmids encoding positive-sense rMP12-GM50 L-, M-, and S-segment downstream of the T7 promoter, designated pProT7-vL(+)-GM50, pProT7-vM(+)-GM50, and pProT7-vS(+)-GM50, respectively, were prepared using a custom DNA synthesis service (GenScript Inc.). Plasmids encoding the negative-sense AMTV M-segment minigenome, pT7-AMTV-M-rLuc(-), or the negative-sense GOLV M-segment minigenome, pT7-GOLV-M-rLuc(-), were prepared using the gBlocks Gene Fragment synthesis service (Integrated DNA Technologies), followed by subcloning into the pT7 plasmid [23]. A plasmid encoding the negative-sense RVFV M-segment minigenome, pT7-RVFV-M-rLuc(-), has been previously described [23]. Plasmids expressing AMTV N, L, or Gn/Gc proteins (i.e., pCAGGS-AMTV-N, pT7-IRES-AMTV-L, or pCAGGS-AMTV-G, respectively) were prepared via custom DNA synthesis (GenScript Inc.) of template DNA, followed by subcloning into the pCAGGS or pT7-IRES plasmid [23]. Plasmids expressing RVFV MP-12 strain N, L, or Gn/Gc proteins (pT7-IRES-vN, pT7-IRES-vL, or pCAGGS-vG, respectively) have been previously described [22].

### Adenovirus vectors

The PCR fragment was amplified from the pENTR4 plasmid (Thermo Fisher Scientific) to remove the chloramphenicol resistant gene and ccdB gene. The resulting PCR fragment containing the attL1 and attL2 sites was recombined with the linearized pCAGGS-vG plasmid cut with *Spe*I and *Xho*I, which encodes the CAG promoter and the entire ORF of RVFV MP-12 M-segment, by Gibson assembly (New England BioLabs). The M-segment ORF of resulting pENTR-CAG-vM plasmid was replaced with N or L ORF of RVFV ZH501 strain; pENTR-CAG-wN or pENTR-CAG-wL, respectively. These pENTR plasmids were further recombined with pAd/PL-DEST Gateway Vector (Thermo Fisher Scientific) by LR clonase according to the manufacture’s instruction. Recombined plasmids lacking resistant genes for both kanamycin and chloramphenicol were selected and linearized with *Pac*I. 293A cells were transfected with linearized DNA, and recombinant human adenovirus 5 vector lacking the entire E1 region, which expresses RVFV N or L proteins, were rescued (rAd5-RVFV-N or rAd5-RVFV-L, respectively).

### Replication kinetics of rMP-12 and rMP12-GM50

C6/36 cells were infected with either the rMP-12 or rMP12-GM50 strain at either 0.01 or 1.0 MOI, at 28°C for 1 hour. After washing cells three times with media, culture supernatant samples were collected at 1 hour post infection (hpi). Cells were further incubated at 28°C, and culture supernatants were collected at 24, 48, 72, 96, and 144 hpi. Three independent experiments were performed.

### Co-infection with rMP-12 and rMP12-GM50 strains, or with rMP-12 and AMTV

C6/36 cells were co-infected with the rMP-12 strain (2 MOI) and rMP12-GM50 strain (3 MOI). Viral titers were determined via plaque assays using Vero cells, and the infectivity of those strains in C6/36 cells were confirmed via indirect fluorescent assay (IFA) (**S1 Fig**). Similarly, C6/36 cells were co-infected with rMP-12 and AMTV, with viral inputs equivalent to 6 and 9 MOI, respectively. At those inputs, most C6/36 cells were infected with rMP-12 and AMTV (**S1 Fig**).After washing five times with media, cells were further incubated at 28°C and then culture supernatants were collected at 24 hpi for plaque isolation for genotyping of L-, M-, and S-segments.

### Co-infection with rMP-12 and GOLV

C6/36 cells were mock-infected or infected with the rMP-12 (0.5 MOI) and/or GOLV (8 MOI). Most C6/36 cells were infected with GOLV at 8 MOI as confirmed via IFA, whereas lower input of rMP-12 (0.5 MOI) was used so that the replication of rMP-12 could occur in GOLV-infected cells (**S1 Fig**). Cells were incubated at 28°C without CO_2_ for 1 hour, and washed with media three times. Cells were then incubated at 28°C for 72 hours. Total RNA was extracted at 72 hpi, and culture supernatant was transferred into fresh C6/36 cells and Vero cells. Cells were washed three times at 1 hour post infection, and then, further incubated at 28°C for 72 hours. Total RNA was extracted at 72 hpi, and analyzed by Northern blot.

### Plaque isolation of virus

Vero cells in 10-cm dishes or six-well plates were infected with supernatant from the co-infected cells at 37°C for 1 hour. An overlay containing 0.6% noble agar, 1 x MEM, 5% TPB, penicillin (50 U/ml), and streptomycin (50 μg/ml) was then added [24]. At 72 hpi, a second overlay, containing an additional 0.012% of neutral red solution, was added. At 96 hpi, plaques isolated in wells were slowly aspirated using a 20 – 200ul tip, and then transferred into 1.7-ml Eppendorf tubes containing 200 μl of supplemented DMEM. Plaque isolates (50 μl) were further amplified in Vero cells at 37°C for 5 to 7 days, and total RNA was extracted from infected Vero cells using the TRIzol reagent (Life Technologies) in order to determine the genotypes of reassortants.

### PCR-restriction fragment length polymorphism (RFLP) analysis

The genotyping of L-, M-, and S-segments from plaque isolates was conducted via PCR-RFLP analysis. PCR primers flanking a restriction enzyme site unique to the parental rMP-12 sequence (S- and M-segment PCR fragments: *Bam*HI site; L-segment PCR fragment: *Pst*I site) were prepared (**S2 Fig**). Primer sets were designed for PCR as follows: S-segment PCR–S341F and S764R; M-segment PCR–M19F and M456R; and L-segment PCR–L1846F and L2362R (**S1 Table**). Template first-stranded cDNA was synthesized with random hexamers, using SuperScript II (Life Technologies) according to the manufacturer’s instruction. The PCR reaction (40 cycles) using Phusion High Fidelity DNA polymerase (New England BioLabs) was performed according to the manufacturer’s instruction. PCR fragments were purified using the DNA Clean & Concentrator-5 Kit (Zymo Research), and then digested for 16 hours at 37°C with restriction enzymes–*Bam*HI for S- and M-segment PCR fragments, and *Pst*I for L-segment PCR fragments. The PCR fragment from the rMP-12 S-segment (472 bp) is cut into a 185-bp fragment and a 291-bp fragment by *Bam*HI. The PCR fragment from the rMP-12 M-segment (480 bp) becomes a 306-bp fragment and a 178-bp fragment following digestion by *Bam*HI. Finally, *Pst*I cuts the PCR fragment from the rMP-12 L-segment (562 bp) into a 121-bp fragment and a 445-bp fragment. The S-, M-, and L-segments of rMP12-GM50 do not encode restriction enzyme sites cut with *Bam*HI or *Pst*I. Genotyping was performed for DNA samples via PCR-RFLP, using electrophoresis on 2% agarose gels.

### Genotyping of L-, M-, and S-segments of AMTV and the rMP-12 strain via RT-PCR

Total RNA from Vero cells infected with each plaque isolate was used for first-strand cDNA synthesis with SuperScript II. RT-PCR was performed separately for L-, M-, and S-segment genotyping (**S3 Fig**). A mixture of primers (RVFV-L488F, RVFV-L1037R, AMTV-L4014F, and AMTV-L4966R) was used for the PCR of the L-segment (rMP-12: 570 bp; AMTV: 1004 bp). A different mixture of primers (RVFV-M999F, RVFV-M1556R, AMTV-M1494F, and AMTV-M2433R) was used for PCR of the M-segment (rMP-12: 568 bp; AMTV: 997 bp). Finally, a third mixture of primers (RVFV-S341F, RVFV-S764R, AMTV-S1035F, and AMTV-S1776R) was used for PCR of the S-segment (rMP-12: 472 bp; AMTV: 786 bp) (**S1 Table**).

### Northern blot analysis

Total RNA was extracted from mock-infected or infected cells using the TRIzol reagent. Denatured RNA was separated on 1% denaturing agarose-formaldehyde gels and transferred onto a nylon membrane (Roche Applied Science). Northern blot analysis was performed, as described previously [25], with strand-specific RNA probes to detect negative-sense RNA from RVFV or GOLV [23,25]. RNA probes for GOLV L-, M-, and S-segments were generated from pSPT18-GOLV-L, pSPT18-GOLV-M, or pSPT18-GOLV-S plasmids via *in vitro* transcription using the DIG RNA Labeling Kit (Sigma-Aldrich), according to the manufacturer’s instruction.

### Luciferase assay

BHK/T7-9 cells in 12-well plates were transfected with 0.8 μg of pT7-AMTV-M-rLuc(-), pT7-RVFV-M-rLuc(-), or pT7-GOLV-M-rLuc(-); 0.8 μg of pCAGGS-AMTV-N or pT7-IRES-vN; 0.1 μg of pT7-IRES-AMTV-L or pT7-IRES-vL; and 0.4 μg of pCAGGS-AMTV-G or pCAGGS-vG, using the TransIT-293 Transfection Kit (Mirus Bio). For the transfection control, 0.01 μg of pT7-IRES-fLuc plasmid [23] was also co-transfected in each sample. At 72 hpi, cells were collected and luciferase activity was measured with the Dual-Luciferase Reporter Assay System (Promega Corporation), following the manufacturer’s instruction. The rLuc activity values were divided with the fLuc activity values to analyze the minigenome activities based on the constitutively expressed fLuc protein levels. The resulting rLuc / fLuc values were shown as percentages: i.e., the median value of control samples, which express neither N nor L proteins, was set as 100%. Since the background rLuc activities varied among RVFV, AMTV and GOLV minigenomes, the controls without N and L expression were set for each minigenome species. For the detection of rLuc activity without fLuc activity measurement, *Renilla* Luciferase Assay System (Promega Corporation) was alternatively used.

### Sequence alignment

Genome sequences of AMTV and the MP-12 strain were aligned by the CLC Genomics Workbench 7.5.2, with the following parameters: gap open cost, 10; gap extension cost, 1; and end gap cost, free.

### Statistical analysis

Statistical analyses were conducted using GraphPad Prism 6.05 (GraphPad Software Inc.). Comparisons of virus titers between two groups were analyzed via unpaired t-test at each time point. For the comparisons of multiple groups, such as normalized rLuc values, arithmetic means of log_10_ values were analyzed by one-way ANOVA, followed by Tukey’s multiple comparison test.

### Data availability

The L-, M-, and S-segment sequences of rMP12-GM50 strain were deposited in the GenBank (accession numbers: MF593928, MF593929, and MF593930). The L-, M-, and S-segment sequences of a plaque isolate of AMTV Ar 1286–64 strain from Vero cells, which was used for the cloning of N, L, and M ORF, were also deposited in the GenBank (accession numbers: MF593931, MF593932, and MF593933).

### Ethics statement

Use of the RVFV MP-12 strain, the recombinant MP-12 strains, AMTV, and GOLV was approved by the Institutional Biosafety Committee at UTMB.

## Results

### Functional compatibility of RVFV N and L proteins for replication of AMTV or GOLV M-segment minigenomes

**Fig 1:**
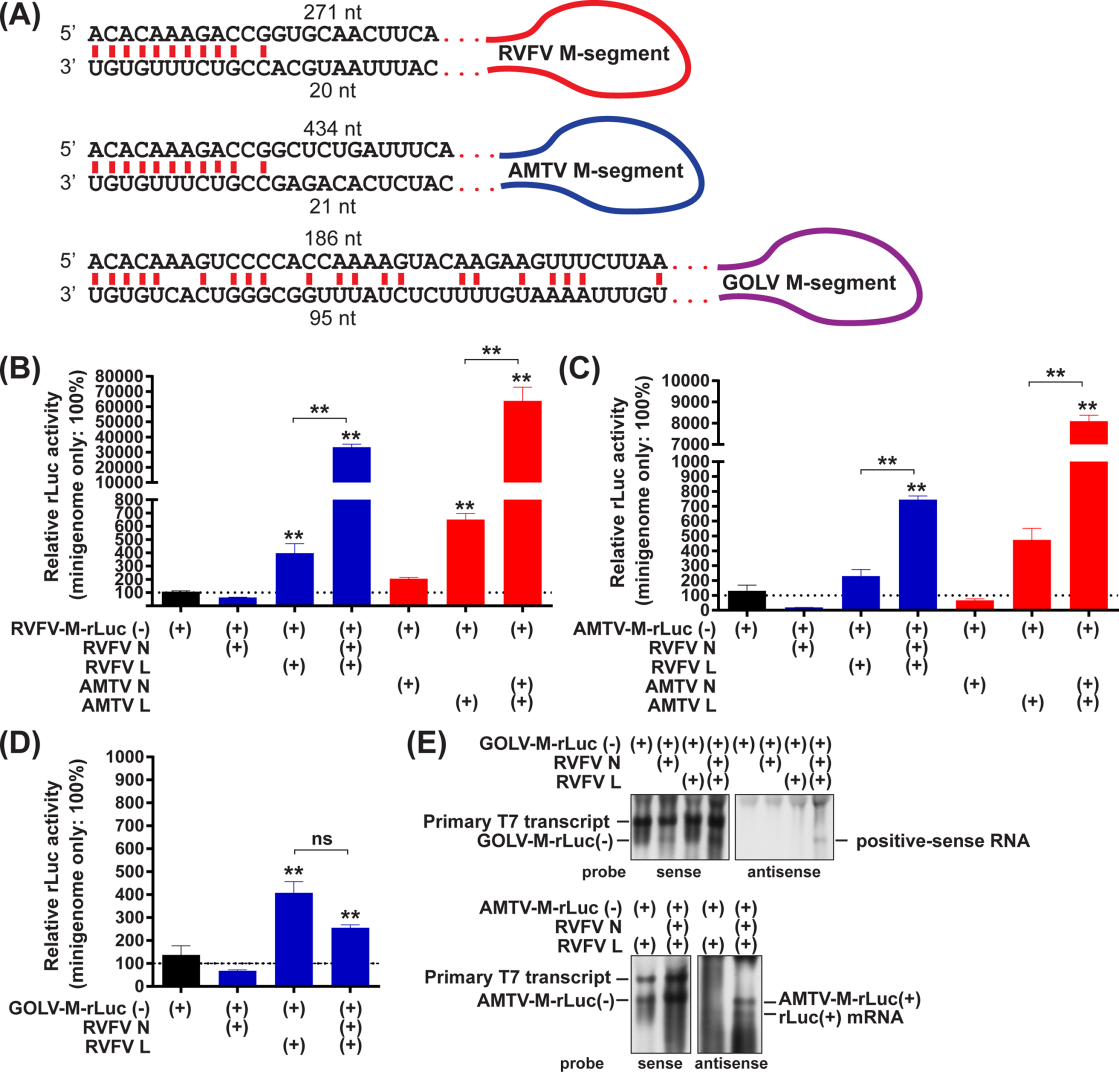
Replication of the M-segment minigenomes of Rift Valley fever phlebovirus (RVFV), Arumowot virus (AMTV), and Gouleako goukovirus (GOLV) in relation to co-expression of RVFV N and L proteins. (A) Schematics of panhandle sequence of M-segments of RVFV, AMTV, and GOLV. (B–D) BHK/T7-9 cells were transfected with plasmids expressing the RVFV-M-rLuc(-) (M-segment minigenome RNA of RVFV) (B), AMTV-M-rLuc(-) (M-segment minigenome RNA of AMTV) (C), or GOLV-M-rLuc(-) (M-segment minigenome RNA of GOLV) (D), and those expressing N and/or L proteins derived from either RVFV or AMTV. The ratio of *Renilla* luciferase (rLuc) activities to firefly luciferase (fLuc) activities derived from pT7-IRES-fLuc (control plasmid) was shown as percentage: i.e., the same type of minigenome without N and L expression was set as 100%. Bars represent means plus standard errors. Asterisks on error bars represent statistically significant increases compared to samples expressing minigenome only (One-way ANOVA **p < 0.01). Samples expressing minigenome, N proteins, and L proteins were also statistically compared with those expressing minigenome, and L proteins. ns, not significant. (E) Northern blot using total RNA derived from the GOLV minigenome assay (top panels) or AMTV minigenome assay (bottom panels). Sense rLuc probe (left panels) and antisense rLuc probe (right panels) were utilized.

Bunyavirus L-, M-, and S-segment RNA are complimentary at the 3’- and 5’-termini, and form panhandle structures that serve as promoters of RNA synthesis and signals for encapsidation with N proteins [26]. Since the panhandle regions are highly conserved among phlebovirus species, it is possible that RVFV N and L proteins support the replication of genomic RNA from heterologous phlebovirus species. M-segment minigenomes, which encode the entire 3’- and 5’-noncoding regions and a single ORF of *Renilla* luciferase (rLuc) in place of the M ORF, were constructed using RVFV, AMTV, and GOLV M-segments (**Fig 1A**). The 3’- and 5’-termini of the RVFV and AMTV M-segments share identical 12-nucleotide sequences, and the panhandle structures could be formed by the terminal 10 nucleotides at the 3’- and 5’-termini. The 3’- and 5’-terminal sequences of the GOLV M-segment encode mismatches at positions six, seven, and nine nucleotides from the termini, which could form a distinct panhandle structure from that formed by the 3’- and 5’-termini of RVFV or AMTV M-segments. To test whether RVFV N and L proteins can support the replication of AMTV or GOLV M-segment minigenomes, BHK/T7-9 cells were transfected with plasmids expressing RVFV N protein, RVFV L protein, and negative-sense minigenome RNA of either the RVFV, AMTV, or GOLV M-segment–RVFV-M-rLuc(-), AMTV-M-rLuc(-), or GOLV-M-rLuc(-), respectively. RVFV N and L proteins increased the rLuc activities of RVFV- M-rLuc(-) and AMTV-M-rLuc(-) by factors of 84 and 3.2, respectively, in comparison to the background rLuc activity in plasmids expressing RVFV L proteins and minigenome RNA without RVFV N proteins (**Fig 1B and 1C**). Similarly, BHK/T7-9 cells were transfected with plasmids expressing AMTV N protein, AMTV L protein, and either RVFV-M-rLuc(-) or AMTV-M-rLuc(-) plasmid. AMTV N and L proteins increased the rLuc activities of RVFV- M-rLuc(-) and AMTV-M-rLuc(-) by factors of 98 and 17, respectively, in comparison to the background rLuc activity in plasmids expressing AMTV L proteins and minigenome RNA without RVFV N proteins (**Fig 1B and 1C**). The results indicated that RVFV N and L proteins support the replication of AMTV M-segment RNA, whereas AMTV N and L proteins support the replication of RVFV M-segment RNA. In contrast to AMTV-M-rLuc(-), RVFV N and L proteins did not increase the rLuc activities of GOLV-M-rLuc(-), indicating that RVFV N and L proteins do not support the replication of GOLV M-segment minigenome (**Fig 1D**). To further characterize the replication of GOLV minigenome via RVFV N and L proteins, total RNAs extracted from transfected cells were further analyzed by Northern blot (**Fig 1E**, top panels). Unexpectedly, cells expressing RVFV N and L proteins showed an accumulation of positive-sense GOLV minigenome-like band. Since it remains unknown whether transcriptional termination occurs in GOLV M-segment via RVFV L protein, it was still possible that rLuc mRNA could be synthesized. A lack of increase in rLuc activity from GOLV minigenome with RVFV N and L proteins (**Fig 1D**) indicates that the presumable rLuc mRNA could be minute in amount, if any. In contrast, Northern blot detected both positive-sense AMTV minigenome RNA band and fast migrating rLuc mRNA band in cells expressing RVFV N protein, RVFV L protein, and AMTV M-segment minigenome (**Fig 1E**, bottom panels). Those results indicated that RVFV L protein could not only synthesize AMTV minigenome RNA, but also recognize the termination signal of AMTV M-segment.

### Co-infection of GOLV and the RVFV MP-12 strain

**Fig 2:**
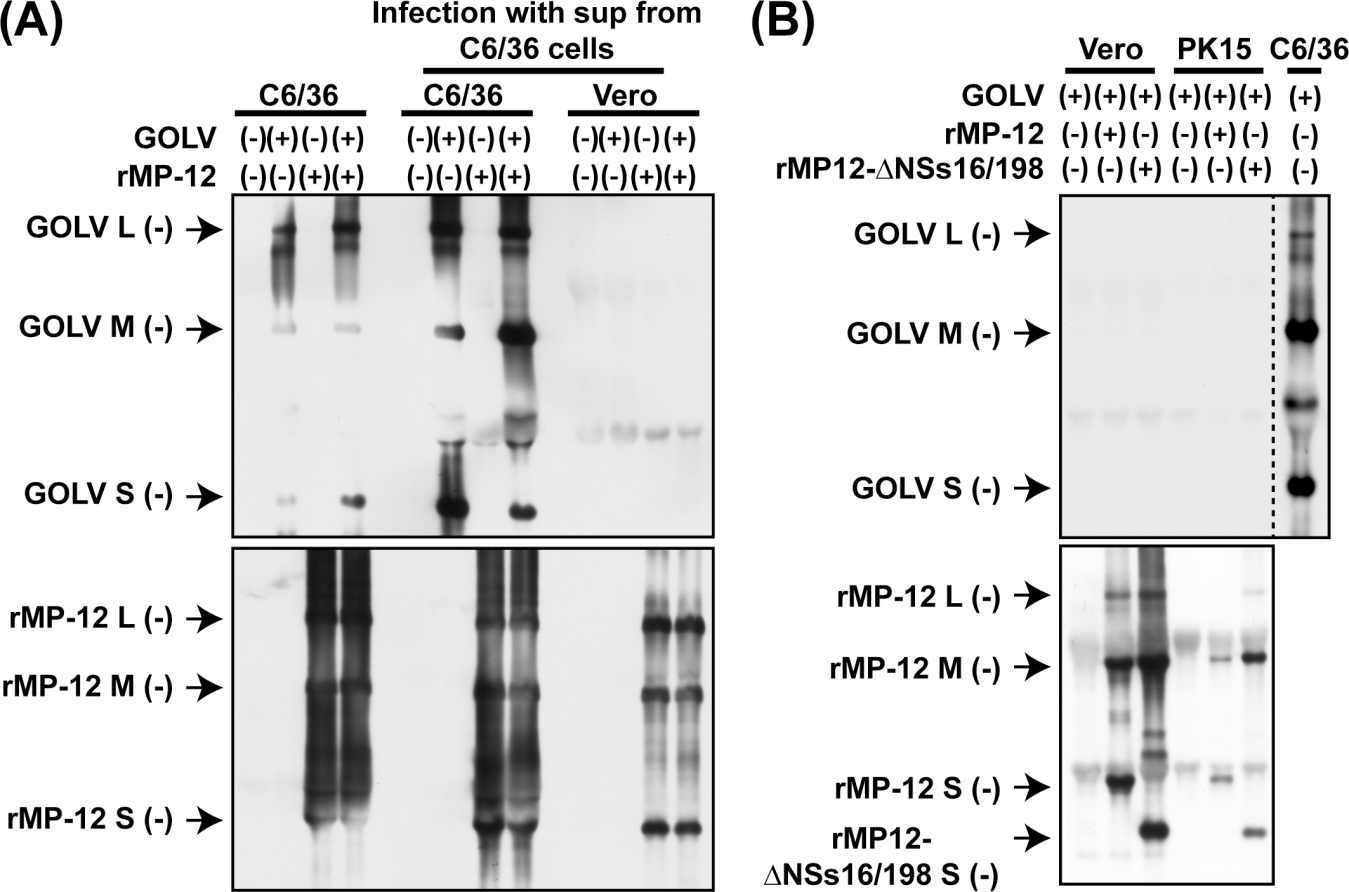
Co-infection of Gouleako goukovirus (GOLV) and RVFV. (A) C6/36 cells were mock-infected or infected with GOLV (8 MOI) and/or rMP-12 (0.5 MOI) at 28°C. Total RNA was extracted at 72 hpi (left four lanes). Culture supernatants were collected and transferred into either C6/36 cells (middle four lanes) or Vero cells (right four lanes). Total RNA was then extracted at 72 hpi. The presence of L-, M-, and S-segment RNA of GOLV (top panel) or RVFV (bottom panel) was analyzed by Northern blot. (B) Vero or PK15 cells were infected with GOLV (8 MOI) with or without either rMP-12 (0.5 MOI) or rMP12-ΔNSs16/198 (2.0 MOI) at 28°C. Total RNA was extracted at 18 hpi, and the accumulation of viral RNA was analyzed by Northern blot. Top panel: probes to detect negative-sense GOLV L-, M-, and S-segments, Bottom panel: probes to detect negative-sense RVFV L-, M-, and S-segments. GOLV RNA derived from C6/36 cells infected with GOLV (8 MOI) was used for the positive control for GOLV probes on the membrane.

GOLV is considered to be a mosquito-restricted bunyavirus: i.e., the RNA replication of GOLV occurs in mosquito cells (*Aedes albopictus*), yet does not occur in culture cells derived from human, nonhuman primate, mouse, hamster, pig, bat, frog, snake, chicken, and fruit fly [17]. GOLV displays a temperature-sensitive phenotype, and viral RNA replication is impaired at 31°C and above in C6/36 cells [18]. It remains unknown whether the infectivity or host specificity of GOLV could be affected via co-infection with RVFV. RVFV N and L proteins could partially support the synthesis of GOLV minigenome RNA in BHK/T7-9 cells (**Fig 1E**). We thus analyzed whether GOLV and MP-12 can form any reassortant strains after co-infection in C6/36 cells. C6/36 cells were mock-infected or infected with GOLV (8 MOI) and/or rMP-12 (0.5 MOI). After infection, total RNA and culture supernatants were collected at 72 hpi. Northern blot analysis showed that GOLV and rMP-12 could synthesize L-, M-, and S-segment RNAs in infected C6/36 cells (**Fig 2A**). Fresh C6/36 cells or Vero cells were subsequently infected with the culture supernatants collected at 72 hpi, to know whether reassortant strains between rMP-12 and GOLV are present at the supernatant and can initiate replication in Vero cells. Cells were incubated at 28°C, and total RNAs were extracted at 72 hpi. Northern blot analysis showed that rMP-12 RNA, but not GOLV RNAs, were detectable in Vero cells, whereas both GOLV and rMP-12 RNAs were detected in similarly infected C6/36 cells (**Fig 2A**). The result demonstrated that GOLV and RVFV do not form reassortant strains which can initiate RNA replication in Vero cells.

GOLV-like virus was isolated from the lung tissues of pigs with severe bronchopneumonia with unknown etiology in the Republic of Korea [19]. Two field strains, CP-1/2013 and CP-12/2013 were subsequently isolated using PK15 cells during the previous study. Although African GOLV (A5/CI/2004 strain) does not replicate in PK15 cells [20], we aimed to know whether the co-infection with RVFV MP-12 strain might affect the replication of GOLV. PK15 cells were co-infected with GOLV (8 MOI) and either rMP-12 (0.5 MOI) or rMP12-ΔNSs16/198 (2 MOI), which encodes an in-frame deletion of NSs gene [22], at 28°C. The rMP12-ΔNSs16/198 was included to evaluate the effect of a lack of functional NSs protein, which block cellular antiviral activities [27,28]. Vero cells were also included in the co-infection experiment for the comparison. At 18 hpi, total RNA was extracted, and the L-, M-, and S-segment RNA of GOLV and MP-12 were detected by Northern blot (**Fig 2B**). The L-, M-, and S-segment RNA of rMP-12 and rMP12-ΔNSs16/198 were accumulated in Vero and PK15 cells, but GOLV L-, M-, and S-segment RNA could not be detected in co-infected cells. The results indicated that GOLV does not initiate viral RNA synthesis in PK15 cells, regardless of co-infection with the MP-12 strain.

As reported previously, GOLV replicated efficiently in C6/36 cells at 28°C, but not at 37°C (**S4 Fig**) [18]. GOLV did not replicate in mammalian cells derived from *Homo sapiens* (MRC-5, A549, or HEC1B cells), *Chlorocebus* sp. (Vero cells), *Sus scrofa* (PK15 cells), *Ovis aries* (OA4.K/S1 cells), and *Mesocricetus auratus* (BHK/T7-9 cells) at 28°C for 120 hours after infection (**S4 Fig**). Taken together, it is unlikely that GOLV forms infectious reassortant strains with RVFV.

### Functional compatibility of RVFV or AMTV N and L proteins for the replication of RNA minigenomes

**Fig 3:**
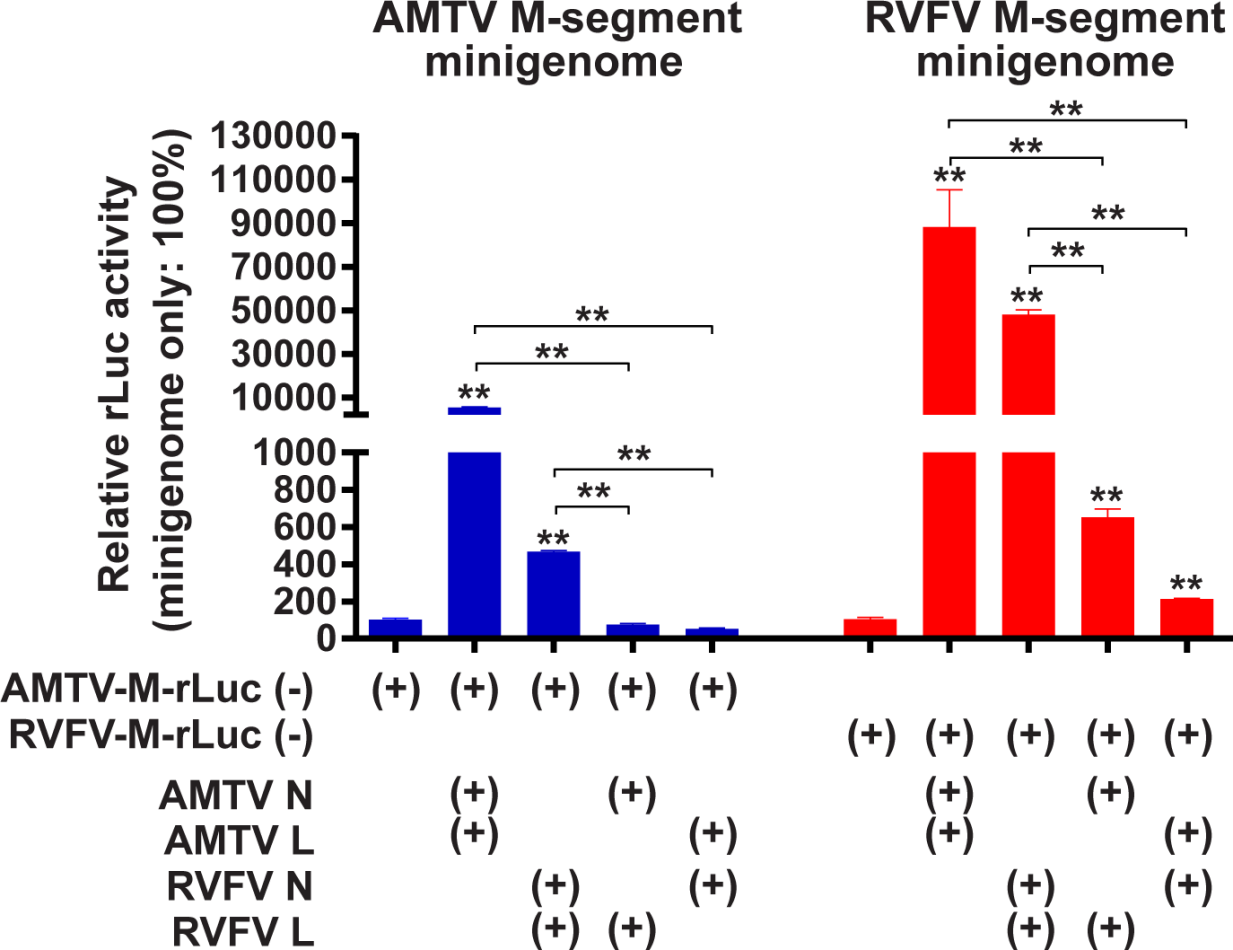
Minigenome assays with expressions of N and L proteins derived from Arumowot virus (AMTV) or Rift Valley fever phlebovirus (RVFV). BHK/T7-9 cells were transfected with plasmids expressing M-segment minigenome RNA from either RVFV or AMTV, and those expressing N or L proteins derived from either AMTV or RVFV. The RVFV-M-rLuc and AMTV-M-rLuc represent the M-segment minigenomes derived from RVFV and AMTV, respectively. Cell lysates were collected at 72 hpt, and the ratio of *Renilla* luciferase (rLuc) activities to firefly luciferase (fLuc) activities derived from pT7-IRES-fLuc (control plasmid) was shown as percentage: i.e., the same type of minigenome without N and L expression was set as 100%. Bars represent means plus standard errors. Asterisks on error bars represent statistically significant increases compared to samples expressing minigenome only (One-way ANOVA **p < 0.01).

Next, we further analyzed the possibility of genetic reassortment between RVFV and AMTV. The amino acid identities between RVFV MP-12 strain (GenBank accession numbers: DQ375404, DQ380208, and DQ380154) and the plaque clone of AMTV Ar 1286–64 strain (GenBank accession numbers: MF593931, MF593932, and MF593933), were as follows: 49% in N protein, 25% in NSs protein, 22% in pre-Gn protein, 30% in Gn protein, 41% in Gc protein, and 57% in L protein. We utilized a minigenome reporter assay to characterize the compatibility of N and L proteins derived from RVFV and AMTV (**Fig 3**). BHK/T7-9 cells were transfected with plasmids expressing minigenome RNA, N protein, or L protein of either RVFV or AMTV, and the rLuc activity was measured at 72 hours post transfection (hpt). The co-expression of AMTV N and RVFV L, or that of RVFV N and AMTV L did not significantly increase the rLuc activities of AMTV-M-rLuc(-) relative to the control sample that lacked N and L protein expression. The co-expression of AMTV N and RVFV L, or that of RVFV N and AMTV L significantly increased the rLuc activities of RVFV- M-rLuc(-) by factors of 6.2 and 2.0, respectively, in comparison to the rLuc activity with plasmids expressing RVFV minigenome RNA without RVFV N and L proteins. Similar increases also occurred when RVFV minigenome RNA and RVFV L protein were expressed (factor of 3.7), or when RVFV minigenome RNA and AMTV L protein were expressed (factor of 6.1) (**Fig 1B**). Comparison of raw luciferase activities however did not show statistically significant differences in fLuc and rLuc values between the samples expressing RVFV L and those expressing AMTV N and RVFV L, or between the samples expressing AMTV L and those expressing RVFV N and AMTV L (**S5 Fig**). These results indicated that AMTV N and RVFV L proteins, or vice versa, do not support the replication of minigenome RNA.

### The ability of RVFV and AMTV Gn/Gc proteins to package minigenome RNA encapsidated with heterogeneous N proteins

**Fig 4:**
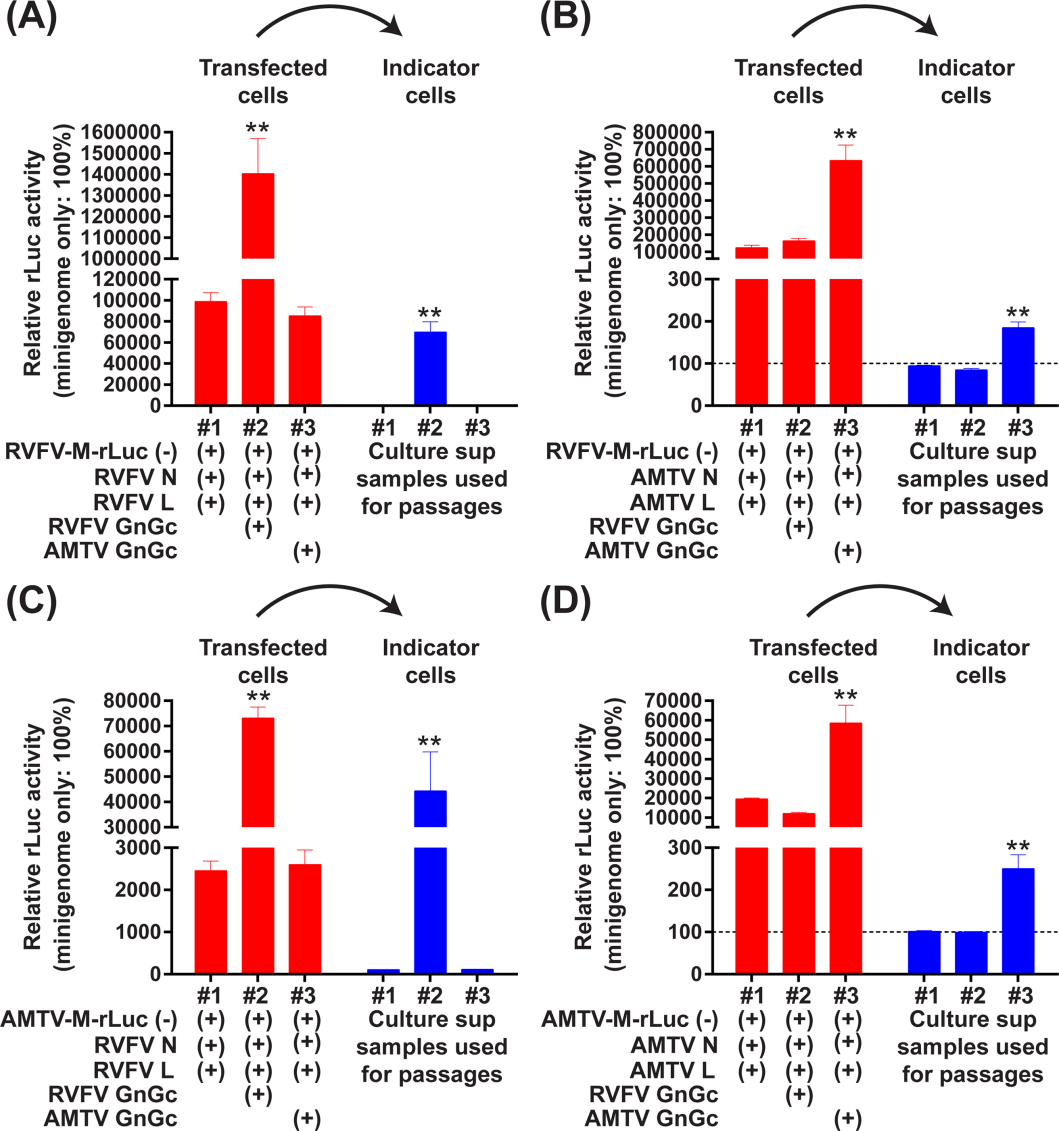
Minigenome assays with expressions of N, L, and Gn/Gc proteins derived from Arumowot virus (AMTV) or Rift Valley fever phlebovirus (RVFV). BHK/T7-9 cells were transfected with plasmids expressing M-segment minigenome RNA from either RVFV (A and B) or AMTV (C and D). Those cells were also co-transfected with plasmids expressing N and/or L derived from either RVFV (A and B) or AMTV (C and D), as well as those expressing Gn/Gc proteins derived from either AMTV or RVFV. The rLuc activity was measured at 72 hpt. Culture supernatants from transfected cells were transferred into fresh Vero cells transduced with adenovirus vectors expressing RVFV N and L proteins for 2 hours immediately before incubation with supernatants. The rLuc activity was then measured 36 hours after incubation with culture supernatants. The ratio of *Renilla* luciferase (rLuc) activities to firefly luciferase (fLuc) activities (transfected cells) or the rLuc activity values (indicator cells) were shown as percentage: i.e., minigenome without N and L expression was set as 100%. Bars represent means plus standard errors. Asterisks on error bars represent statistically significant increases compared to samples expressing minigenome only (One-way ANOVA **p < 0.01).

Next, the ability of Gn/Gc proteins to package minigenome RNA, which is encapsidated with heterologous N proteins, was evaluated by the production of infectious virus-like particles (VLP) carrying the RVFV-M-rLuc(-) minigenome (**Fig 4**). BHK/T7-9 cells were transfected with plasmids encoding the RVFV-M-rLuc(-) or AMTV-M-rLuc(-) minigenomes, as well as plasmids expressing N, L, and Gn/Gc proteins from either RVFV or AMTV. Vero cells that were transduced with recombinant adenoviruses expressing either RVFV N or L proteins were incubated with culture supernatant samples from BHK/T7-9 cells, which might contain VLP, at 37°C for 1 hour. The rLuc activity of Vero cell lysates was then analyzed at 36 hours post VLP infection. The rLuc activity derived from RVFV-M-rLuc(-) minigenome was significantly increased in indicator Vero cells infected with VLP samples derived from transfected cells expressing RVFV N, RVFV L, and RVFV Gn/Gc proteins (**Fig 4A**), and in those expressing AMTV N, AMTV L, and AMTV Gn/Gc proteins (**Fig 4B**). Similarly, the rLuc activity derived from AMTV-M-rLuc(-) minigenome was significantly increased in Vero cells infected with VLP samples derived from cells expressing RVFV N, RVFV L, and RVFV Gn/Gc proteins (**Fig 4C**), and in those expressing AMTV N, AMTV L, and AMTV Gn/Gc proteins (**Fig 4D**).The rLuc activities were not, however, increased in cells incubated with culture supernatants derived from cells expressing RVFV N, RVFV L, and AMTV Gn/Gc proteins, or in those expressing AMTV N, AMTV L, and RVFV Gn/Gc proteins. The results indicated that RVFV Gn/Gc proteins are not capable of packaging the minigenome encapsidated with AMTV N and L proteins, and vice versa. It was noted that rLuc activities were also significantly increased in transfected cells, when cells expressed either the combination of RVFV N, RVFV L, and RVFV Gn/Gc proteins, or that of AMTV N, AMTV L, and AMTV Gn/Gc proteins. The result indicated that spread of VLP also occurred in transfected cells.

### Analysis of reassortant formation between rMP-12 and rMP12-GM50, and between rMP-12 and AMTV in C6/36 cells

**Fig 5:**
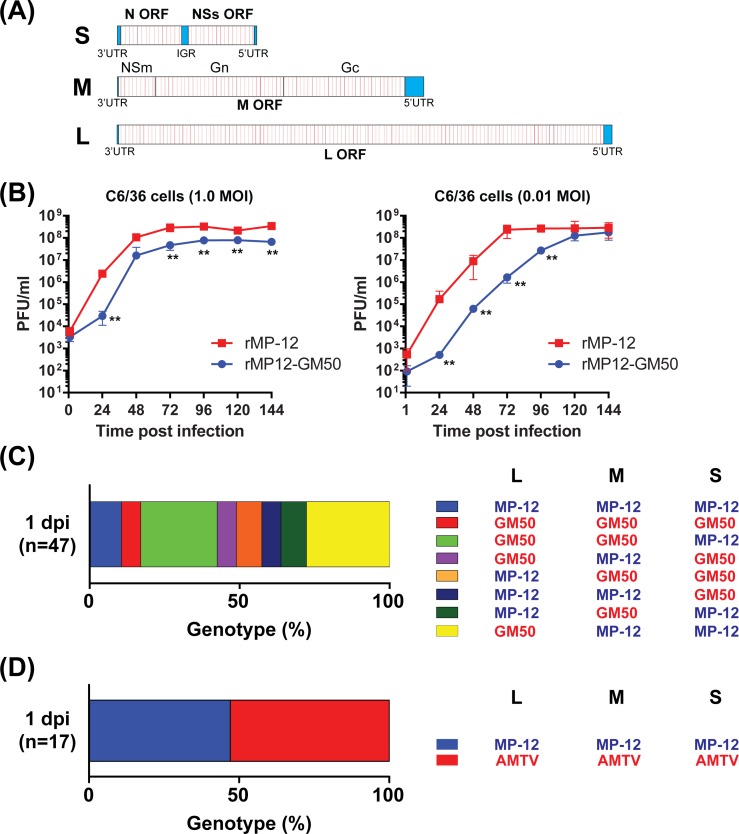
Reassortant formation between rMP-12 and rMP12-GM50 strains, or between rMP-12 and Arumowot virus (AMTV). (A) The schematics of the rMP12-GM50 L-, M-, and S-segments. The rMP12-GM50 strain encodes 326, 185, and 73 silent mutations in the L-, M-, and S-segments, respectively. Individual silent mutations are shown in red. (B) Replication kinetics of rMP-12 and rMP12-GM50 strains in culture cells. C6/36 cells were infected with rMP-12 or rMP12-GM50 (0.01 MOI), and viral titers in culture supernatants were measured via plaque assay. The graph represents the mean ± standard deviation of three independent experiments. Statistical differences between the two strains were analyzed by unpaired t-test at each time point (**p < 0.01). (C) Genetic reassortants in C6/36 cells co-infected with rMP-12 and rMP12-GM50 strains at 24 hours post infection (hpi). Genotypes for L-, M-, and S-segments of 47 plaque isolates from co-infected C6/36 cells were determined via PCR-RFLP analysis (**S2 Fig**). (D) Genetic reassortants in C6/36 cells co-infected with rMP-12 and AMTV at 24 hpi. Genotypes for L-, M-, and S-segments of 17 plaque isolates from co-infected C6/36 cells were determined via RT-PCR analysis (**S3 Fig**).

The occurrence of genetic reassortment between MP-12 and AMTV was further analyzed via a co-infection assay using C6/36 cells. The condition of reassortment was initially evaluated via co-infection with two different MP-12 strains. To analyze reassortant formation within the same species, we newly generated a genetic variant of the MP-12 strain, rMP12-GM50, which encodes a cluster of silent mutations every 50 nucleotides in the N, NSs, M, and L ORFs (**Fig 5A:** red lines), causing 326 mutations in the L-segment, 185 mutations in the M-segment, and 73 mutations in the S-segment. Although both rMP-12 and rMP12-GM50 could replicate up to similar virus titers (10^7^ to 10^8^ PFU/ml) in C6/36 cells, rMP12-GM50 was slightly delayed in reaching plateau titers (**Fig 5B**).

For the reassortment assay, C6/36 cells were co-infected with parental recombinant MP-12 (rMP-12) and rMP12-GM50. Virus inputs were adjusted so that all C6/36 cells were infected. The culture supernatants were collected at 24 hpi, and a total of 47 plaques were cloned from the culture supernatants. Vero cells were infected with each plaque clone, and total RNA from infected Vero cells was extracted for the genotyping of L, M-, and S-segments by PCR-RFLP (**S2 Fig**). Genotyping of L-, M-, and S-segments showed that 10.6% of the plaque clones was parental rMP-12, 6.4% was parental rMP12-GM50, and the remaining 83% of plaque clones belonged to reassortant strains. More specifically, 25.5% were G/G/M reassortants, 6.4% were G/M/G, 8.5% were M/G/G, 6.4% were M/M/G, 8.5% were M/G/M, and 27.7% were G/M/M reassortants (M = rMP-12, G = rMP12-GM50, order = L/M/S) (**Fig 5C**). The results showed that co-infection of C6/36 cells with two different MP-12 strain variants can generate all combinations of reassortant strains at 24 hpi.

Next, the occurrence of genetic reassortment between the RVFV MP-12 strain and AMTV was determined using a similar co-infection assay using C6/36 cells. Randomly-selected seventeen well-isolated viral plaques were collected and amplified once in Vero cells (**S3 Fig**). RVFV and AMTV could not be distinguished based on plaque phenotypes. Total RNA was extracted from infected Vero cells, and the genotypes of L-, M-, and S-segments were determined by RT-PCR assay (**S3 Fig**). As a result, eight clones (47%) encoded the L-, M-, and S-segments of rMP-12, and nine clones (53%) encoded those of AMTV, but no reassortant strains were identified among plaque isolates (**Fig 5D**).

## Discussion

Vaccination protects livestock from severe diseases caused by pathogenic RVFV strains, as well as preventing subsequent transmission of RVFV strains to mosquitoes. Freire et al. showed that the genetic diversity of RVFV strains has steadily decreased since the massive vaccination of ruminants with live-attenuated Smithburn vaccine that occurred in the 1970s [29,30]. Genetic reassortment, however, not only occurs between the same species, but is also known to incorporate RNA segments from other species to create novel pathogenic bunyavirus strains. The Ngari virus (unclassified orthobunyavirus within the family *Peribunyaviridae*), a reassortant strain consisting of Bunyamwera virus L- and S-segments, and the Batai virus M-segment (64% amino acid identity with Bunyamwera virus), was isolated from hemorrhagic fever patients during an RVF outbreak in East Africa in 1997–1998 [9,31]. It is therefore important to characterize the potential genetic exchange between RVFV and other mosquito-borne bunyaviruses for risk management of RVF vaccines used in endemic areas. Within *Phenuiviridae* family, AMTV, ODRV, and GOLV are transmitted by mosquitoes in Africa [13,16,17]. In Burkina Faso, sheep showed serological evidence of infection with either AMTV, ODRV, or RVFV [12]. AMTV induces transient fever in experimentally infected sheep [15], and shares vector mosquitoes (*Culex antennatus*) with RVFV [13]. Little is known concerning the ability of RVFV to incorporate other phlebovirus RNA segments through the reassortment process. Previously, the RNA synthesis compatibility of RVFV and sandfly-borne TOSV N and L proteins was characterized via a minigenome assay [32]. The study showed that the co-expression of RVFV N and L proteins, or of TOSV N and L proteins, supports the expression of chloramphenicol acetyltransferase (CAT) activity from the S-like minigenome of RVFV or the TOSV backbone. Interestingly, the co-expression of TOSV N and RVFV L also increased CAT activity from the RVFV minigenome, but not from the TOSV minigenome. The co-expression of RVFV N and TOSV L did not, however, increase CAT activity from both the RVFV and TOSV minigenomes. The amino acid identities between the RVFV MP-12 strain and TOSV (11368 strain, GenBank Accession KU925898 and KU925899) were 49% for N protein and 54% for L protein (data not shown), even lower than the identities between RVFV and AMTV. Our results showed, however, that the co-expression of RVFV N and AMTV L proteins, or that of AMTV N and RVFV L proteins, does not support minigenome replication. Moreover, the minigenome encapsidated with RVFV N and L proteins cannot be packaged into AMTV Gn/Gc proteins, and vice versa. Taken together, it can be concluded that the genetic reassortant strains between RVFV and AMTV are not viable, due to the incompatibility of N, L, and Gn/Gc proteins. Although this study focused on AMTV for the risk analysis of genetic reassortment with RVFV, similar approach will be applicable to characterize the genetic compatibility of ODRV or other phleboviruses in the future.

GOLV is a mosquito-restricted bunyavirus that is distantly related to RVFV within the *Phenuiviridae* family. Although GOLV is not known to replicate in mammalian cells [17,18], a GOLV-like virus was isolated from the lung tissues of dead pigs, using PK15 cells, in the Republic of Korea [19,20]. It is thus a potential concern that GOLV might alter the replication capability during the co-infection with RVFV. As shown in this study, however, GOLV did not initiate viral replication in PK15 or Vero cells at 28°C, regardless of co-infection with the rMP-12 or rMP12-ΔNSs16/198 strains. Moreover, culture supernatants from C6/36 cells co-infected with rMP-12 and GOLV did not contain infectious reassortant strains, which could initiate viral replication in Vero cells. It is thus unlikely that RVFV can form reassortant strains by incorporating the GOLV RNA segments. Since the genetic difference between RVFV and GOLV is larger than that between RVFV and AMTV [17], their respective N, L, and Gn/Gc proteins are likely not compatible with one another. Unexpectedly, a positive-sense RNA band could be detected from GOLV minigenome in the presence of both RVFV N and L proteins. Since the rLuc activity from GOLV minigenome was not significantly increased via the co-expression of RVFV N and L proteins, it was unlikely that RVFV N and L proteins support the mRNA transcription from GOLV minigenome. Overall, it is unlikely that GOLV will become a public health concern through the formation of infectious reassortant strains with RVFV in Africa.

To understand the generation of reassortant strains in co-infected cells, we generated an MP-12 strain variant, rMP12-GM50, which encodes 584 silent mutations in the ORFs of the L-, M-, and S-segments. This pattern of silent mutations is not found in natural RVFV isolates, and the nucleotide difference is larger than the genetic diversity found among natural RVFV strains (4 to 4.5%) [33]. This strain is thus useful not only for characterizing genetic reassortment within a RVFV species, but also for studies of homologous recombination of RVFV strains. PCR-RFLP analysis showed that samples from co-infected C6/36 cells at 24 hpi contain all possible combinations of L-, M-, and S-segment reassortants between the rMP-12 and rMP12-GM50 strains, indicating that there is little bias when generating reassortant strains in co-infected C6/36 cells. Therefore, genetic reassortment between live-attenuated vaccine strains and pathogenic RVFV strains could be a concern [30]. The MP-12 strain, however, induces a low level (10^2.5^ pfu/ml) of viremia in ruminants, which is considered not sufficient to orally infect mosquitoes in laboratories [34,35]. Moreover, the MP-12 strain is attenuated through point mutations introduced in the L-, M-, and S-segments; therefore, any reassortant strain encoding one or two MP-12 RNA segments will be more attenuated than the pathogenic parental RVFV strains [36]. Although the MP-12 vaccine strain has not been approved for vaccine use in Africa, this well-characterized strain will likely be useful for future vaccination of livestock in endemic countries.

## Conclusions

Genetic reassortment between the MP-12 strain and other pathogenic RVFV strains will likely occur if cells are co-infected with two viruses. The MP-12 strain cannot, however, form reassortant strains with AMTV or GOLV, due to virological incompatibility for replication.

## Supporting information

S1 Fig Indirect fluorescent assay (IFA) to test the infectivity of rMP-12, Arumowot virus (AMTV), or Gouleako goukovirus (GOLV) in C6/36 cells. C6/36 cells were fixed with methanol at 8 hpi, and incubated with 1 in 800 dilution of either anti-RVFV mouse ascites, anti-RVFV N rabbit polyclonal antibody [Won S, Ikegami T, Peters CJ, Makino S (2006) NSm and 78-kilodalton proteins of Rift Valley fever virus are nonessential for viral replication in cell culture. *J*. *Virol*. 80: 8274–8278.], anti-GOLV N (peptide: N-AKK IAE KST PET KRW LES MIQ KYS-C) rabbit custom antibody (ProSci Inc.), or anti-AMTV mouse ascites. Secondary antibodies (1 in 800 dilutions) of either Alexa Fluor 488 goat anti-mouse IgG (H+L), Alexa Fluor 488 goat anti-rabbit IgG (H+L), or Alexa Fluor 594 goat anti-rabbit IgG (H+L) were utilized for the detection of specific signals. (A) mock-infected controls, (B) no primary antibody controls, (C) C6/36 cells infected with rMP-12 at 2 or 1 MOI, (D) C6/36 cells infected with rMP12-GM50 at 3 or 1.5 MOI, (E) C6/36 cells co-infected with GOLV (8 MOI) and rMP-12 (0.5 MOI), and (F) C6/36 cells co-infected with AMTV (9 MOI) and rMP-12 (6 MOI). Nuclei were stained with 4,6-diamidino-2-phenylindole (DAPI) (blue). Scale bars represent 50 μm. RVFV or GOLV antigens were abundantly detected in cytoplasm, whereas AMTV antigens were detected less abundantly due to the weak reactivity of anti-AMTV mouse ascites.
(JPG)

S2 Fig PCR-RFLP analysis to genotype L-, M-, and S-segments of rMP-12 and rMP12-GM50 strains. To genotype the L-, M-, and S-segments, we set up PCR primers flanking a *Bam*HI site unique to the MP-12 S- or M-segments, or a *Pst*I site unique to the MP-12 L-segment. To validate the PCR-RFLP, we used reverse genetics to generate reassortant rMP-12 strains encoding either the S-, M-, or L-segment of rMP12-GM50 (rMP12-RST-GM50-S, rMP12-RST-GM50-M, or rMP12-RST-GM50-L, respectively). Vero cells were infected with either rMP-12, rMP12-GM50, rMP12-RST-GM50-S, rMP12-RST-GM50-M, or rMP12-RST-GM50-L at 1 MOI. Total RNA was collected from infected cells at 24 hpi, and then cDNA was synthesized using random hexamers. PCR-RFLP analysis on agarose gel showed the expected sizes of bands corresponding to the genotype in each segment.
(JPG)

S3 Fig Analysis of genetic reassortment between the RVFV rMP-12 strain and Arumowot virus (AMTV). (A) C6/36 cells were co-infected with rMP-12 (6 MOI) and AMTV (9 MOI) at 28°C for 1 hour. After washing, cells were further incubated at 28°C for 24 hours. A total of 17 plaques were cloned from the 24 hpi supernatant and then re-amplified once in Vero cells. (B) Total RNA from infected Vero cells was used for RT-PCR to determine the genotypes of the L-, M-, and S-segment of each clone. PCR was performed separately for the L-segment (a mixture of RVFV-L488F, RVFV-L1037R, AMTV-L4014F, and AMTV-L4966R), M-segment (a mixture of RVFV-M999F, RVFV-M1556R, AMTV-M1494F, and AMTV-M2433R), and S-segment (a mixture of RVFV-S341F, RVFV-S764R, AMTV-S1035F, and AMTV-S1776R). The plasmids pProT7-vL, pProT7-vM, and pProT7-vS were used as positive controls for the RVFV L-, M-, and S-segments, respectively. Similarly, the plasmids pProT7-AMTV-L, pProT7-AMTV-M, and pProT7-AMTV-S were used as controls for the AMTV L-, M-, and S-segments, respectively.
(JPG)

S4 Fig Gouleako goukovirus (GOLV) infection in C6/36 and mammalian cells. (A) Formation of GOLV focus in C6/36 cells at 28°C under 0.3% tragacanth gum at 72 hours post infection (hpi). Cells were fixed with 100% methanol and stained with anti-GOLV N rabbit polyclonal antibody, followed by Alexa Fluor 594 goat anti-rabbit IgG (H+L) secondary antibody (Thermo Fisher Scientific). (B) C6/36 cells were infected with GOLV at 0.01 MOI at 28°C or 37°C, and washed three times with media. Cells were incubated at 28°C or 37°C, and culture supernatants were collected at 1, 24, 48, 72, and 96 hpi. The graph represents the mean + standard errors of virus titers from triplicated samples. (C) MRC-5, A549, HEC1B, Vero, PK15, OA4.K/S1, or BHK/T7-9 cells were infected with GOLV at 0.01 MOI at 28°C. Cells were incubated at 28°C and culture supernatants were collected at 1, 72, and 120 hpi. Cells were verified to be mycoplasma free (UTMB Tissue Culture Core Facility), and the identity of MRC-5, A549, and HEC1B cells were authenticated by Short Tandem Repeat analysis (UTMB Molecular Genomics Core Facility). The graph represents the mean + standard errors of virus titers from triplicated samples.
(JPG)

S5 Fig Raw luciferase activity values from minigenome assays with expressions of N and L proteins derived from Arumowot virus (AMTV) or Rift Valley fever phlebovirus (RVFV). (A) BHK/T7-9 cells were transfected with plasmids expressing M-segment minigenome RNA from either RVFV or AMTV, and those expressing N or L proteins derived from either AMTV or RVFV. The RVFV-M-rLuc and AMTV-M-rLuc represent the M-segment minigenomes derived from RVFV and AMTV, respectively. Cell lysates were collected at 72 hpt, and *Renilla* luciferase (rLuc) activity values and firefly luciferase (fLuc) activity values are separately shown in the graph. The original fLuc value was divided with 100 so that the rLuc and fLuc values could be shown at a similar scale. Bars represent means plus standard errors. One-way ANOVA: ns, not significant.
(JPG)

S1 Table Sequences of primers used for the genotyping of L-, M-, and S-segments of rMP-12 and rMP12-GM50 strains. Sequences of primers used for RT-PCR of L-, M-, and S-segments are listed.
(DOCX)

## Abbreviations

AMTV: Arumowot virus
CAT: chloramphenicol acetyltransferase
fLuc: firefly luciferase
GFV: Gabek Forest virus
GOLV: Gouleako goukovirus
GORV: Gordil virus
hpi: hour post infection
hpt: hour post transfection
ODRV: Odrenisrou virus
ORF: open reading frame
PCR-RFLP: PCR-Restriction Fragment Length Polymorphism
PNV: Punique virus
rLuc: *Renilla* luciferase
RVF: Rift Valley fever
RVFV: Rift Valley fever phlebovirus
SAFV: Saint Floris virus
SFSV: Sandfly fever Sicilian virus
SNSV: Sandfly fever Naples virus
TOSV: Toscana virus
VLP: virus-like particle
