# Supplementary figures and images for: Risk analysis of inter-species reassortment through a Rift Valley fever phlebovirus MP-12 vaccine strain
Source: PLoS One. 2017 Sep 19;12(9):e0185194. doi: 10.1371/journal.pone.0185194 (PMC5604998; doi:10.1371/journal.pone.0185194)

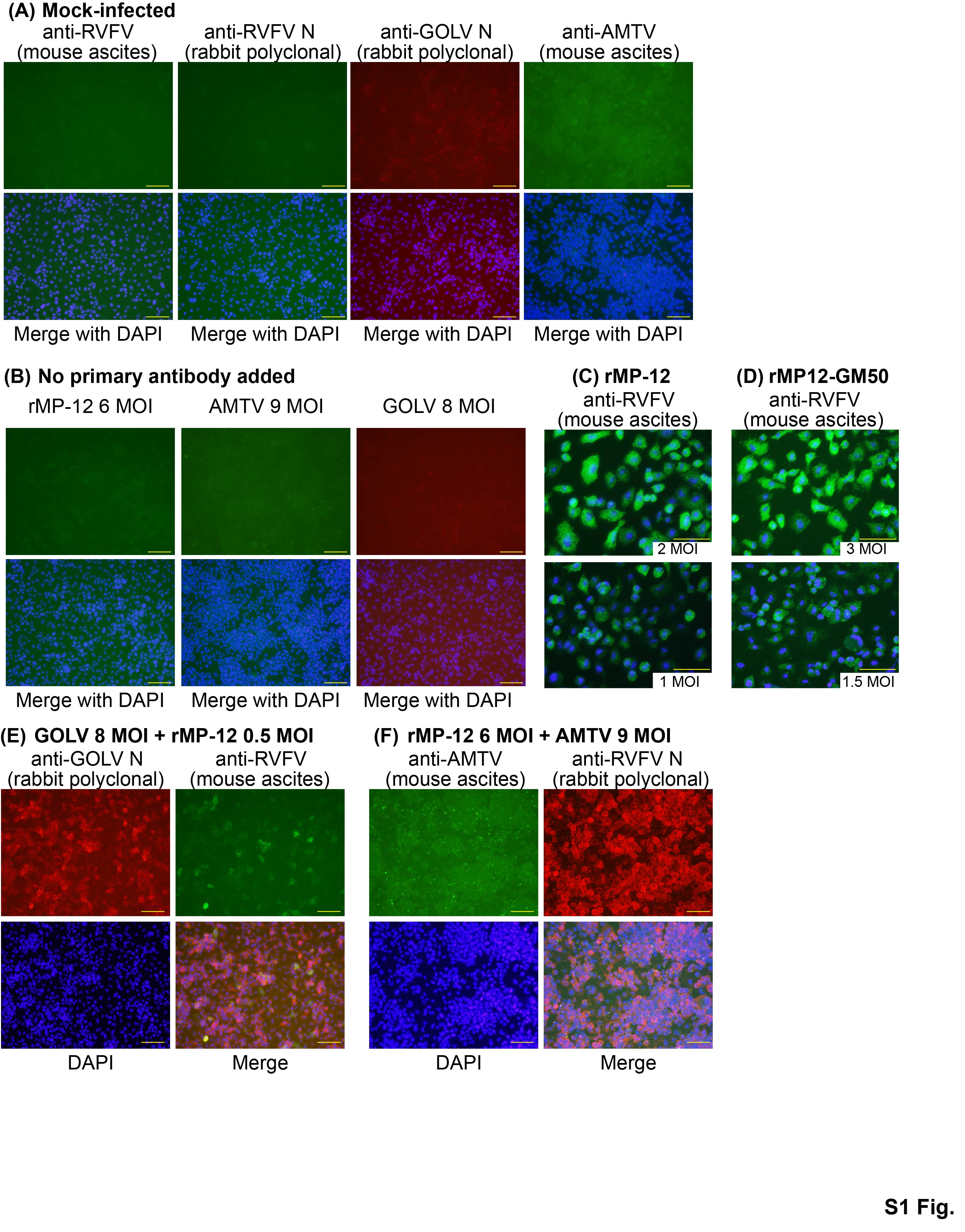

Supplement: S1 Fig — C6/36 cells were fixed with methanol at 8 hpi, and incubated with 1 in 800 dilution of either anti-RVFV mouse ascites, anti-RVFV N rabbit polyclonal antibody [Won S, Ikegami T, Peters CJ, Makino S (2006) NSm and 78-kilodalton proteins of Rift Valley fever virus are nonessential for viral replication in cell culture. J. Virol. 80: 8274–8278.], anti-GOLV N (peptide: N-AKK IAE KST PET KRW LES MIQ KYS-C) rabbit custom antibody (ProSci Inc.), or anti-AMTV mouse ascites. Secondary antibodies (1 in 800 dilutions) of either Alexa Fluor 488 goat anti-mouse IgG (H+L), Alexa Fluor 488 goat anti-rabbit IgG (H+L), or Alexa Fluor 594 goat anti-rabbit IgG (H+L) were utilized for the detection of specific signals. (A) mock-infected controls, (B) no primary antibody controls, (C) C6/36 cells infected with rMP-12 at 2 or 1 MOI, (D) C6/36 cells infected with rMP12-GM50 at 3 or 1.5 MOI, (E) C6/36 cells co-infected with GOLV (8 MOI) and rMP-12 (0.5 MOI), and (F) C6/36 cells co-infected with AMTV (9 MOI) and rMP-12 (6 MOI). Nuclei were stained with 4,6-diamidino-2-phenylindole (DAPI) (blue). Scale bars represent 50 μm. RVFV or GOLV antigens were abundantly detected in cytoplasm, whereas AMTV antigens were detected less abundantly due to the weak reactivity of anti-AMTV mouse ascites. (JPG) [file pone.0185194.s001.jpg]

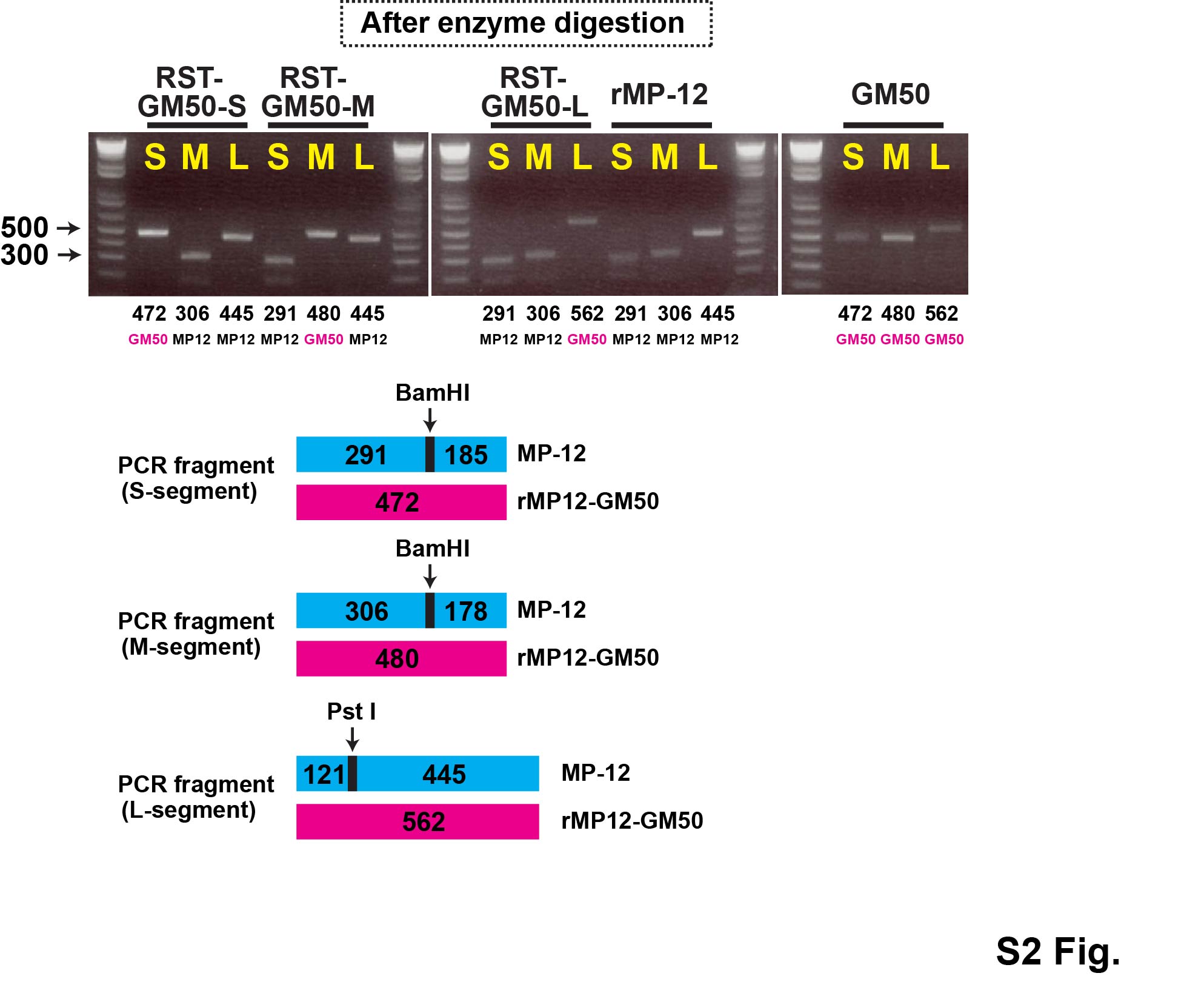

Supplement: S2 Fig — To genotype the L-, M-, and S-segments, we set up PCR primers flanking a BamHI site unique to the MP-12 S- or M-segments, or a PstI site unique to the MP-12 L-segment. To validate the PCR-RFLP, we used reverse genetics to generate reassortant rMP-12 strains encoding either the S-, M-, or L-segment of rMP12-GM50 (rMP12-RST-GM50-S, rMP12-RST-GM50-M, or rMP12-RST-GM50-L, respectively). Vero cells were infected with either rMP-12, rMP12-GM50, rMP12-RST-GM50-S, rMP12-RST-GM50-M, or rMP12-RST-GM50-L at 1 MOI. Total RNA was collected from infected cells at 24 hpi, and then cDNA was synthesized using random hexamers. PCR-RFLP analysis on agarose gel showed the expected sizes of bands corresponding to the genotype in each segment. (JPG) [file pone.0185194.s002.jpg]

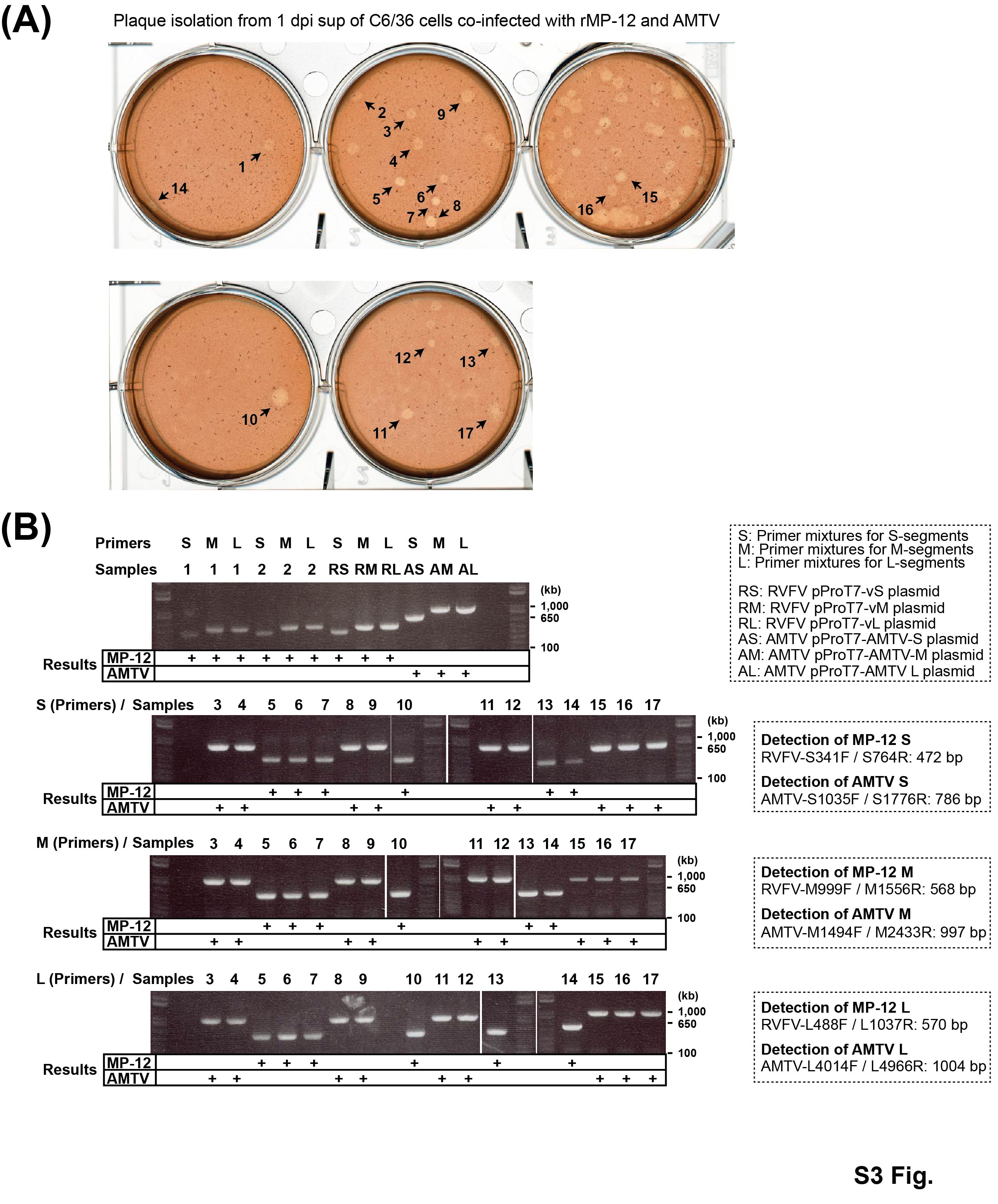

Supplement: S3 Fig — (A) C6/36 cells were co-infected with rMP-12 (6 MOI) and AMTV (9 MOI) at 28°C for 1 hour. After washing, cells were further incubated at 28°C for 24 hours. A total of 17 plaques were cloned from the 24 hpi supernatant and then re-amplified once in Vero cells. (B) Total RNA from infected Vero cells was used for RT-PCR to determine the genotypes of the L-, M-, and S-segment of each clone. PCR was performed separately for the L-segment (a mixture of RVFV-L488F, RVFV-L1037R, AMTV-L4014F, and AMTV-L4966R), M-segment (a mixture of RVFV-M999F, RVFV-M1556R, AMTV-M1494F, and AMTV-M2433R), and S-segment (a mixture of RVFV-S341F, RVFV-S764R, AMTV-S1035F, and AMTV-S1776R). The plasmids pProT7-vL, pProT7-vM, and pProT7-vS were used as positive controls for the RVFV L-, M-, and S-segments, respectively. Similarly, the plasmids pProT7-AMTV-L, pProT7-AMTV-M, and pProT7-AMTV-S were used as controls for the AMTV L-, M-, and S-segments, respectively. (JPG) [file pone.0185194.s003.jpg]

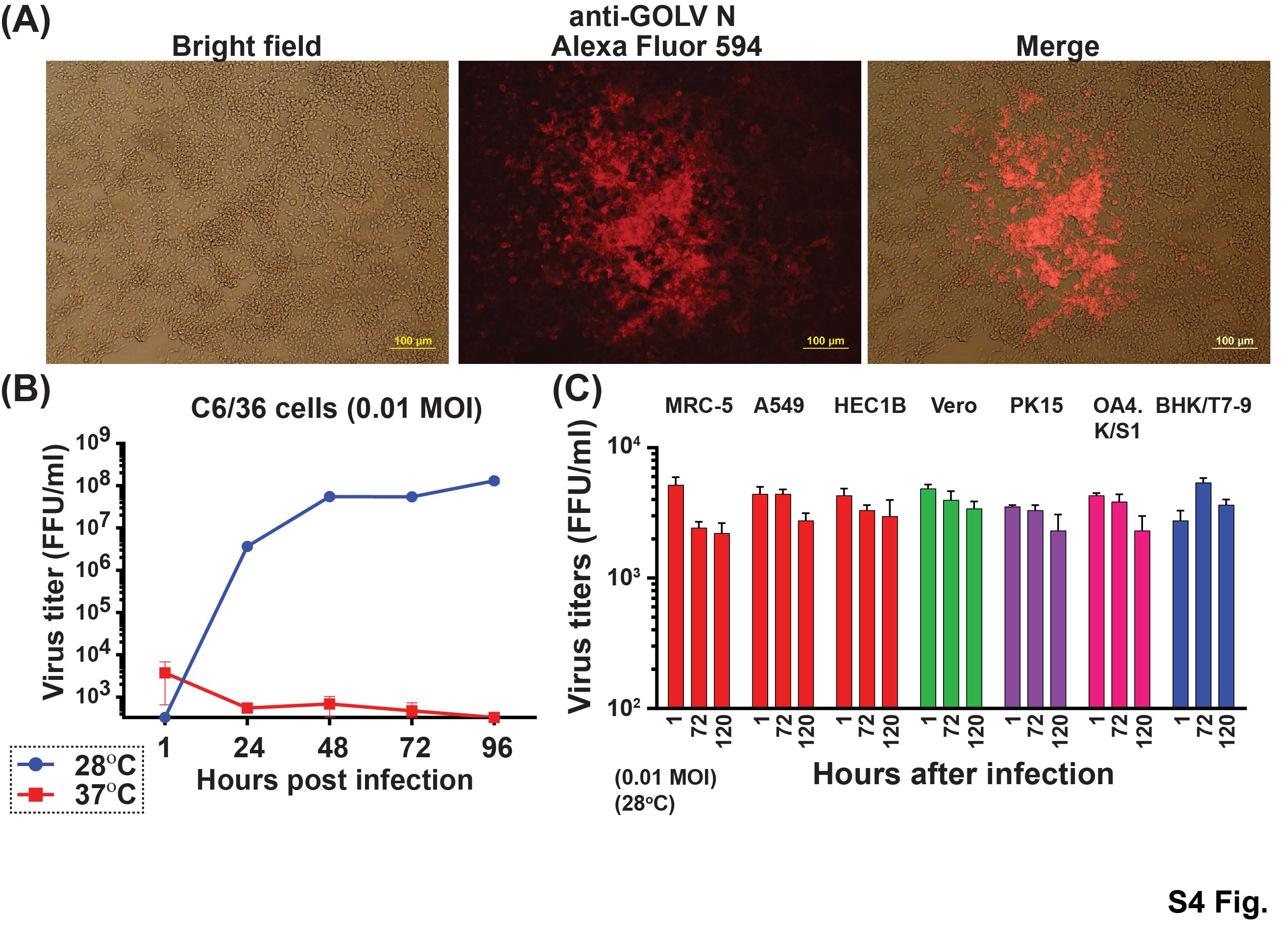

Supplement: S4 Fig — (A) Formation of GOLV focus in C6/36 cells at 28°C under 0.3% tragacanth gum at 72 hours post infection (hpi). Cells were fixed with 100% methanol and stained with anti-GOLV N rabbit polyclonal antibody, followed by Alexa Fluor 594 goat anti-rabbit IgG (H+L) secondary antibody (Thermo Fisher Scientific). (B) C6/36 cells were infected with GOLV at 0.01 MOI at 28°C or 37°C, and washed three times with media. Cells were incubated at 28°C or 37°C, and culture supernatants were collected at 1, 24, 48, 72, and 96 hpi. The graph represents the mean + standard errors of virus titers from triplicated samples. (C) MRC-5, A549, HEC1B, Vero, PK15, OA4.K/S1, or BHK/T7-9 cells were infected with GOLV at 0.01 MOI at 28°C. Cells were incubated at 28°C and culture supernatants were collected at 1, 72, and 120 hpi. Cells were verified to be mycoplasma free (UTMB Tissue Culture Core Facility), and the identity of MRC-5, A549, and HEC1B cells were authenticated by Short Tandem Repeat analysis (UTMB Molecular Genomics Core Facility). The graph represents the mean + standard errors of virus titers from triplicated samples. (JPG) [file pone.0185194.s004.jpg]

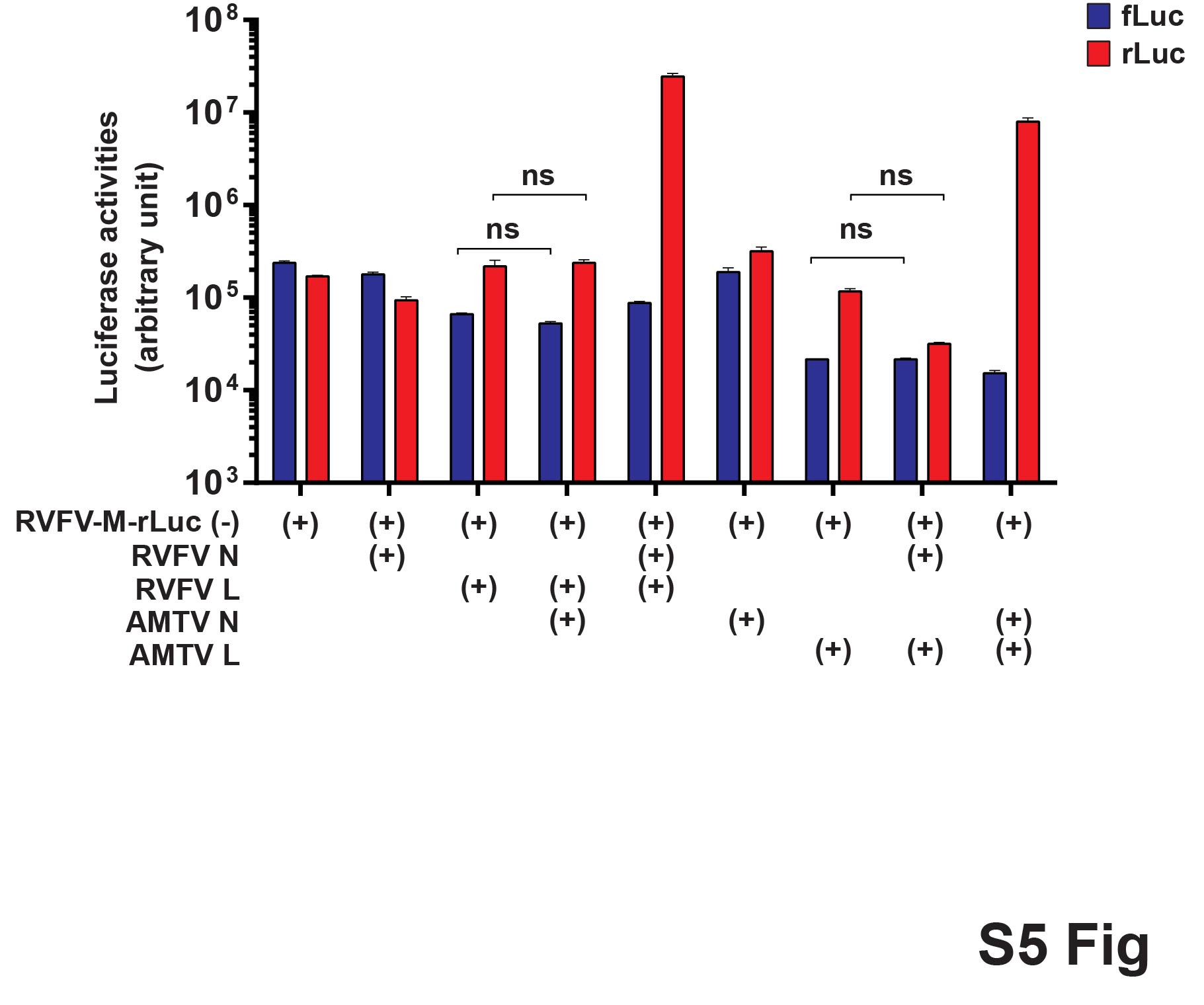

Supplement: S5 Fig — (A) BHK/T7-9 cells were transfected with plasmids expressing M-segment minigenome RNA from either RVFV or AMTV, and those expressing N or L proteins derived from either AMTV or RVFV. The RVFV-M-rLuc and AMTV-M-rLuc represent the M-segment minigenomes derived from RVFV and AMTV, respectively. Cell lysates were collected at 72 hpt, and Renilla luciferase (rLuc) activity values and firefly luciferase (fLuc) activity values are separately shown in the graph. The original fLuc value was divided with 100 so that the rLuc and fLuc values could be shown at a similar scale. Bars represent means plus standard errors. One-way ANOVA: ns, not significant. (JPG) [file pone.0185194.s005.jpg]
