# Supplementary material for: Risk analysis of inter-species reassortment through a Rift Valley fever phlebovirus MP-12 vaccine strain
Source: PLoS One. 2017 Sep 19;12(9):e0185194. doi: 10.1371/journal.pone.0185194 (PMC5604998; doi:10.1371/journal.pone.0185194)
Supplement: S1 Table — Sequences of primers used for RT-PCR of L-, M-, and S-segments are listed. (DOCX) [file pone.0185194.s006.docx]

**Supplementary Table 1. Primers used for the genotyping of L-, M-, and S-segments**

| Primer Name | Target | | Sequence |
| --- | --- | --- | --- |
|  | |  |  |
| **Genotyping: rMP-12 vs. rMP12-GM50** | | | |
| S341F | | MP-12 S | 5’- GGA AGG GAA TCC TTC CCG GGA TGA G -3 |
| S764R | | MP-12 S | 5’- CTG GGC AGC CAC TTA GGC TGC TGT C -3’ |
| M19F | | MP-12 M | 5’- ACA CAA AGA CGG TGC ATT A -3’ |
| M456R | | MP-12 M | 5’- CAG CAA AAA CAA CAG GTG CCA AAG C -3 |
| L1846F | | MP-12 L | 5’- GCT TAC CAA CCT CTG CAA GTG CG -3’ |
| L2362R | | MP-12 L | 5’- CTG TGG TCT AAG GTG CTC AAG TTC -3’ |
|  | |  |  |
| **Genotyping: rMP-12 vs. AMTV** | | |  |
| RVFV-S341F | | MP-12 S | 5’- GGA AGG GAA TCC TTC CCG GGA TGA G -3’ |
| RVFV-S764R | | MP-12 S | 5’- CTG GGC AGC CAC TTA GGC TGC TGT C -3’ |
| AMTV-S1035F | | AMTV S | 5’- CAG CAA TGA TGT TGA CAT TGC TC -3’ |
| AMTV-S1776R | | AMTV S | 5’- ACA CAA AGA CCC CTG AAA GAG TT -3’ |
| RVFV-M999F | | MP-12 M | 5’- CAA GCA CTC AAA AAG TGT GAT GG -3’ |
| RVFV-M1556R | | MP-12 M | 5’- GCG ATC CTG TGA CGC AAA CTC CGC-3’ |
| AMTV-M1494F | | AMTV M | 5’- CTT GTG TTG CGA AGT GTC ATC AGA ATT CC -3’ |
| AMTV-M2433R | | AMTV M | 5’- GAC ACA GTG GAA GCA TTC CAC TCT AAG CAG -3’ |
| RVFV-L488F | | MP-12 L | 5’- GCA GGA CTG TTG TTC TTT ACG TTG -3’ |
| RVFV-L1037R | | MP-12 L | 5’- GTC CCT GAA GAG GGC ACA GAT CTT -3’ |
| AMTV-L4014F | | AMTV L | 5’- ACC AGA TGA TCC AAA TGA GAT TCC TG -3’ |
| AMTV-L4966R | | AMTV L | 5’- GGC AAA ATC TGG CTT TTG TCT CGT CTG -3’ |
|  | |  |  |
|  | | | |
|  | | | |
